# Nanoplastics and fungi: exploring dual roles in degradation and pathogenicity

**DOI:** 10.3389/fmicb.2025.1679160

**Published:** 2025-10-08

**Authors:** Yuanyuan Ma, Yifan Zhou, Dongnan Zheng, Wenxia Bu, Fengxu Wang, Xinyuan Zhao, Peng Xue

**Affiliations:** Department of Occupational Medicine and Environmental Toxicology, Nantong Key Laboratory of Environmental Toxicology, School of Public Health, Nantong University, Nantong, China

**Keywords:** nanoplastics, fungi, pathogenicity, antifungal resistance, plastic degradation

## Abstract

Plastic pollution, particularly in the form of nanoplastics, represents a growing global environmental crisis with profound impacts on ecosystems and human health. This review investigates the multifaceted interactions between fungi and nanoplastics, highlighting fungi’s dual role in both the degradation of plastics and their potential pathogenicity. Fungi possess specialized enzymatic pathways, which empower them to effectively break down a variety of plastic materials, leading to innovative bioremediation approaches. However, the omnipresence of nanoplastics in the environment poses significant challenges, as they can adversely affect fungal physiology, altering metabolic processes, enhancing virulence, and potentially contributing to antifungal resistance. This review examines the mechanisms through which different fungal species degrade specific plastics while emphasizing the influence of nanoplastics on fungal metabolic pathways and collective community dynamics. It explores the adaptations fungi may exhibit in response to nanoplastic exposure, including changes in enzymatic activity and resistance mechanisms. Additionally, the review addresses the implications of nanoplastic exposure for the pathogenicity of fungi, particularly concerning their interactions with human hosts and resistance to antifungal treatments. By providing a thorough analysis of the current understanding of nanoplastics and fungi, this review calls for urgent research into the ecological consequences of these interactions and the potential for increasing antifungal resistance. Ultimately, this work aims to inform effective strategies for mitigating the dual threats of plastic pollution and fungal-related health issues.

## 1 Introduction

Plastic pollution has emerged as a critical global environmental challenge, significantly affecting ecosystem health, human wellbeing, and long-term sustainability (Wilcox et al., 2015; Geyer et al., 2017; Hoseini and Bond, 2022; Cowger et al., 2024). The degradation of plastic materials occurs through various mechanisms, including UV irradiation, thermal degradation, mechanical stress, and biodegradation. These processes ultimately lead to the formation of microplastics and nanoplastics (Fotopoulou and Karapanagioti, 2019; Zhang et al., 2021). Microplastics, defined as plastic particles smaller than 5 mm, and nanoplastics, defined as particles smaller than 100 nm, have garnered significant attention in recent years (Kong et al., 2025; Sun et al., 2025). Nanoplastics, in particular, are concerning due to their ubiquitous presence in the environment and their potential harmful effects on living organisms. Their unique physicochemical properties enhance both their reactivity and bioavailability, raising severe environmental and health concerns (Gigault et al., 2021; Mitrano et al., 2021; Sui et al., 2023; Bu et al., 2025).

Nanoplastics are not only a pollutant but also an emerging factor influencing microbial communities in various ecosystems (Nath et al., 2020; Ren et al., 2022; Zhao et al., 2025). They interact with a diverse array of microorganisms, including bacteria, archaea, and fungi, potentially altering their physiological functions and ecological roles. For instance, studies have demonstrated that exposure to polystyrene nanoplastics leads to a decrease in both bacterial and fungal biomass within microfabricated soil models (Mafla-Endara et al., 2023). Interestingly, some bacterial species have demonstrated the ability to either degrade nanoplastics or utilize them as a substrate. This interaction can significantly affect microbial community dynamics and nutrient cycling in aquatic environments (Wang et al., 2023). Such changes are crucial, as they may influence the biogeochemical processes that underpin ecosystem services. Fungi, as essential microorganisms, play vital roles in nutrient cycling and overall ecosystem functioning. Certain fungal species have shown remarkable abilities to degrade various plastics, making them promising candidates for bioremediation strategies (Rodrigues et al., 2019; Ryu et al., 2020). Their significance in plastic biodegradation is attributed to their capacity to secrete a diverse array of degrading enzymes, which catalyze the conversion of complex plastic polymers into simpler, more manageable compounds. This enzymatic activity facilitates the oxidation or hydrolysis of plastics, resulting in the formation of functional groups that increase hydrophilicity. Consequently, high molecular weight plastics can be transformed into lower molecular weight compounds that fungi can readily assimilate (Temporiti et al., 2022; Ibrahim et al., 2024; Yang et al., 2024). Some fungi exhibit the ability to effectively degrade specific plastics within just a few days, underscoring their efficiency in addressing plastic pollution (Ibrahim et al., 2024).

Despite their beneficial potential, it is essential to recognize that fungi are not exclusively advantageous. Invasive fungal species contribute to an estimated 2.5 million human fatalities each year (Denning, 2024). Among these, *Cryptococcus neoformans*, *Aspergillus fumigatus*, *Candida albicans*, and *Candida auris* have been identified by the World Health Organization (WHO) as some of the most dangerous pathogenic fungi to humans (Kriegl et al., 2024). As research increasingly investigates the interactions between fungi and nanoplastics, it becomes crucial to evaluate how these plastic nanoparticles impact fungal physiology and pathogenicity. The adsorption of nanoplastics onto fungal cell surfaces may lead to alterations in physiological functions, potentially affecting ecological roles and interactions with other organisms. Furthermore, the presence of nanoplastics may influence the metabolic pathways of fungi, leading to changes in their enzymatic profiles and nutrient assimilation capabilities. This could result in both positive and negative outcomes, depending on the specific context and species involved. For example, while some fungi might enhance their plastic-degrading capabilities in response to nanoplastic exposure, others may experience detrimental effects that impair their metabolic functions and ecological interactions. The ability of fungi to adapt to the presence of nanoplastics raises important questions concerning their potential virulence and antifungal resistance, particularly in human pathogenic species. The alterations in fungal physiology due to nanoplastic interactions could lead to an increased expression of virulence factors or enhanced resistance mechanisms against antifungal agents. Understanding these dynamics is crucial for developing effective strategies to combat both plastic pollution and fungal infections.

This review aims to provide a comprehensive examination of the relationships between nanoplastics and fungi by systematically analyzing the mechanisms of plastic degradation by fungi, their metabolic pathways, and physiological adaptations. Additionally, it will address the potential implications for virulence and antifungal resistance in human pathogenic fungi. By elucidating these complex interactions, this review seeks to contribute to the broader understanding of how nanoplastics impact fungal communities and their ecological roles, as well as to inform strategies aimed at mitigating the dual threats of plastic pollution and fungal diseases.

## 2 The role of fungi in plastic degradation

**Figure 1:**
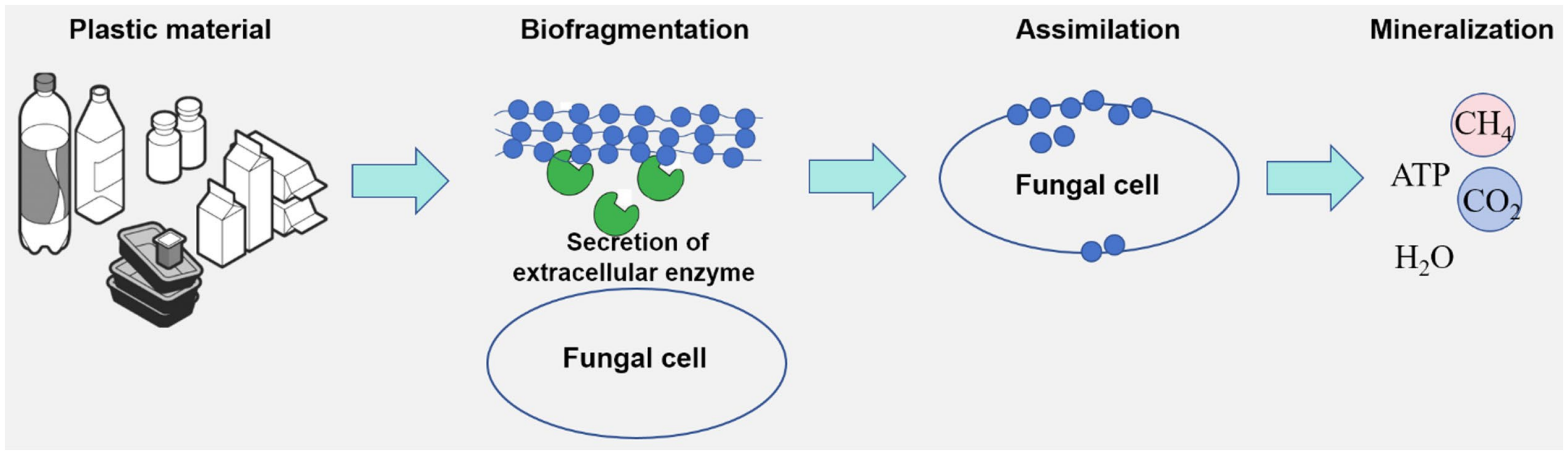
Schematics of fungal degradation process.

Fungi play a pivotal role in the degradation and assimilation of various plastic materials (Figure 1), including polyethylene, polystyrene, and polyvinyl chloride (Maity et al., 2021; Cowan et al., 2022; Vaksmaa et al., 2023). Certain fungal species have demonstrated the remarkable capacity to degrade specific plastics within a matter of days, highlighting their potential for addressing plastic pollution (Okal et al., 2023).

The advantages of fungal degradation can be summarized into three key points:

### 2.1 Enhanced substrate penetration

The apical growth pattern of filamentous fungal mycelia enables these organisms to penetrate soil substrates more effectively than bacteria. This capability is attributed to their secretion of extracellular enzymes and hydrophobic proteins, which enhance their adhesion to hydrophobic substrates (Sánchez, 2020). Such penetration is particularly pronounced in soil environments where saprotrophic fungi thrive (Dix et al., 1995).

### 2.2 Diverse enzymatic machinery

Fungi possess a wide range of non-specific enzyme cassettes (Aranda, 2016; Deshmukh et al., 2016), which equip them to degrade and utilize hydrocarbons and aromatic compounds found in plastics as carbon and energy sources. Secreted fungal enzymes, including lignin peroxidase, manganese peroxidase, laccase, and polyesterase (Table 1), facilitate the oxidation or hydrolysis of plastic polymers, leading to the formation of functional groups that increase hydrophilicity. This enhanced hydrophilicity promotes the breakdown of high molecular weight plastics into lower molecular weight compounds that fungi can readily assimilate. Additionally, various internal factors within fungal cells can induce pro-oxidant ions, which promote degradation through oxidative reactions, particularly through the generation of reactive oxygen species. Hydroxyl radicals, recognized for their high redox potential, are particularly effective oxidants in this context (Backa et al., 1993; Lawton and Robertson, 1999). This oxidative mechanism enables fungi to participate in the degradation of a variety of recalcitrant pollutants, including hydrocarbons, chlorophenols, and pesticides (Young et al., 2015; Deshmukh et al., 2016; Hasan, 2018).

**Table 1:** Key fungal groups and their enzymatic roles in plastic degradation.

Genus	Representative species	Enzymes	Features	References
*Ganoderma*	*Ganoderma lucidum*; *G. applanatum*	Lignin peroxidase, manganese peroxidase, laccase, polyesterase	Degradation of plastics and other organic materials	Mir-Tutusaus et al. (2018)
*Pleurotus*	*Pleurotus abalones*; *P. ostreatus*	Cellulases, lignin peroxidase, manganese peroxidase, laccase	Capable of degrading polylactic acid (PLA), polyethylene (PE), polypropylene (PP), and polyester plastics such as PET	Espinosa-Valdemar et al. (2011); Odigbo et al. (2023)
*Penicillium*	*Penicillium chrysogenum*; *P. funiculosum*; *P. simplicissimum*;	Polyesterases, esterases, ligninases, cellulases, phenol oxidase	Degradation of plastics such as polyurethane and polyethylene	Wolski et al. (2012); Ogunmolu et al. (2015); Sowmya et al. (2015)
*Aspergillus*	*Aspergillus niger; A. oryzae; A. fumigatus*	Esterases, polyesterases, cellulases, phenol oxidase, ligninases	Capable of degrading polyester plastics, such as PET	Maeda et al. (2005); El-Dash et al. (2023); Safdar et al. (2024)
*Mucor*	*Mucor circinelloides; M. hiemalis*	Polyesterases, esterases, oxidases, cellulases	Capable of degrading polyester plastics	Marchut-Mikolajczyk et al. (2015); Al Mousa et al. (2022)
*Trametes*	*Trametes versicolor; T. hirsuta*	Lignin peroxidase, manganese peroxidase, laccase, polyesterase	Effective in plastic degradation, especially for polymers like polyurethane and polyethylene	Arora and Sandhu (1984); Vršanská et al. (2017)
*Phanerochaete*	*Phanerochaete chrysosporium*	Lignin peroxidase, manganese peroxidase, laccase, polyesterase	Degradation of lignin and certain plastics	Kersten and Cullen (2007)
*Lentinus*	*Lentinus edodes; L. tigrinus*	Lignin peroxidase, manganese peroxidase, laccase, cellulase	Effective in degrading lignin and some synthetic materials	Covino et al. (2010); Zavarzina et al. (2018); Kobayashi et al. (2023)
*Cladosporium*	*Cladosporium* sp. P7	Cutinase (CpCut1)	Polyurethane degradation	Liu et al. (2024)
*Alternaria*	*Alternaria alternata* FB1	Peroxidase, laccase	Polyurethane degradation	Gao et al. (2022)
*Penicillium*	*P. citrinum*	Laccase, lipase, esterase, manganese peroxidase	Degradation of low density polyethylene	Khan et al. (2023)
*Malassezia*	*Malassezia* species	Depolymerase, lipase	Degradation of Polyvinyl Chloride	El-Dash et al. (2023)
*Cladosporium*	*Cladosporium* sp. CPEF-6	Laccase	Degradation of low density polyethylene	Gong et al. (2023)
*Clonostachys*	*Clonostachys rosea*	Cutinases	Degradation of polycaprolactone	Gambarini et al. (2025)

### 2.3 Adaptability to diverse environment

Fungi exhibit remarkable adaptability, enabling them to utilize nearly any organic carbon source and thrive in conditions characterized by low humidity, nutrient scarcity, and acidic pH levels (Case et al., 2025). Some species can endure dry periods through a state of cryptobiosis (Magan, 2007; Mancera-López et al., 2008). The metabolic pathways within fungi are mediated by cytochrome P450 (CYP) monooxygenases (EC:1.14.13.12), which facilitate the oxidation of substrates in microsomes (Durairaj et al., 2015; Asemoloye et al., 2019). These enzymes belong to the heme-protein superfamily and are involved in various biological processes, including adaptation to environmental stress, toxin production, and the metabolism of both endogenous and xenobiotic compounds, thereby enhancing fungal fitness (Bernhardt, 2006; Van Beilen and Funhoff, 2007; Kelly et al., 2009; Moktali et al., 2012). CYP monooxygenases typically function as terminal oxidases in the electron transfer chain associated with NADPH reductase, facilitating the incorporation of an oxygen atom into the hydrocarbon chain of nanoplastics while reducing the other oxygen atom to water (Chen et al., 2014). The number of CYP genes varies according to the lifestyle of the fungal species; yeasts and yeast-like fungi possess relatively few CYPs (e.g., three in *Saccharomyces cerevisiae*, six in *C. neoformans*, and 10 in *C. albicans*), while filamentous fungi typically harbor a greater number of CYP genes, as exemplified by *Aspergillus* spp. with 79 genes and *Agaricus bisporus* with 109 (Doddapaneni et al., 2013; Dauda et al., 2022). As a result, plastic-degrading fungi are predominantly filamentous species (Table 1), including *Ganoderma lucidum*, *Pleurotus abalone*, *Penicillium chrysogenum*, and *Aspergillus niger* (Wolski et al., 2012; Mir-Tutusaus et al., 2018; Odigbo et al., 2023; Safdar et al., 2024). Ekanayaka et al. assessed 395 filamentous fungal strains from the Ascomycota and Basidiomycota phyla, identifying over 200 species capable of degrading various plastics under diverse environmental conditions (Ekanayaka et al., 2022). Their findings revealed that fungi such as *Aspergillus tubingensis* effectively disrupt the chemical bonds within plastic molecules and successfully colonize plastic surfaces (Ekanayaka et al., 2022). Numerous plastic-degrading fungi have been isolated from both terrestrial and marine environments (Viel et al., 2023), including *Trichoderma* sp., *Clitocybe* sp., *Monascus* sp., and *Phanerochaete* sp., which enhance the degradation of polyethylene (both LDPE and HDPE), polylactic acid, polyurethanes, polyethylene terephthalate, and bisphenol A polycarbonate (Artham and Doble, 2010; El-Morsy et al., 2017; Ojha et al., 2017; Satti et al., 2017; Janczak et al., 2018; Munir et al., 2018). Marine environments have yielded marine yeasts such as *Rhodotorula mucilaginosa*, *Zalerion maritimum*, *Alternaria alternata*, *Penicillium* spp., and *Aspergillus* sp., which significantly facilitate the degradation of polyethylene and polystyrene, contributing to healthier ecosystems by reducing plastic waste (Ameen et al., 2015; Sarkhel et al., 2020; Gao et al., 2022; Vaksmaa et al., 2023; do Paço, A.M.S., 2024). Overall, the capacity of fungi to degrade plastics presents a promising avenue for bioremediation strategies aimed at mitigating the environmental impacts of plastic waste. Advances in the exploration of fungal enzymes, along with genetic engineering techniques, could enhance biodegradation processes and contribute to sustainable waste management practices.

### 2.4 The impact of nanoplastics on fungal physiology and pathogenicity

Nanoplastics possess unique physical properties, including an increased surface area, specific transport characteristics, and distinctive interactions with light and natural colloids (Gigault et al., 2021). The larger surface area enhances the adsorption capacity of nanoplastics for natural organic matter in the environment (Liu et al., 2022). Adsorption predominantly occurs through chemical bonding on certain types of nanoplastics, facilitated by ligand exchange mechanisms with oxide nanoplastics. This interaction reduces surface hydrophobicity, increases interactions among plastic particles, and affects their aggregate size (Junaid and Wang, 2021). Furthermore, the presence of electron-attracting groups within the aromatic rings of nanoplastic polymers facilitates strong *π*–π interactions, contributing to their exceptional ion adsorption properties (Hüffer and Hofmann, 2016; Wang et al., 2020). The substances adsorbed onto nanoplastics can interact with extracellular polymers secreted by fungal cells, potentially enveloping the nanoplastics in a unique layered structure referred to as the eco-corona. This eco-corona can significantly alter the dynamics between nanoplastics and fungi (Liu et al., 2022). Fungal cell wall thickness typically ranges from 0.1 to 1.0 micrometers, and the formation of an ecological corona layer on these walls is contingent upon the abundance and physicochemical properties of biomolecules and plastic particles. The stability of this layer is influenced by hydrogen bonds, van der Waals forces, hydrophobic interactions, and other high-energy chemical or adhesive forces (Liu et al., 2022). Research indicates that the zeta potential of fungal cell walls is highly responsive to environmental conditions, generally fluctuating between −14 and −15 millivolts (Ramos et al., 2020). Changes in environmental pH, along with varying concentrations of ions and proteins, can promote heteroaggregation, which may consequently alter the zeta potential (Mikolajczyk et al., 2015). Exposure of fungal cells to nanoplastics may modulate the zeta potential of their cell walls, thereby affecting their functional integrity and potentially contributing to the toxicity of extracellularly secreted enzymes.

The fungal cell wall serves as the outermost layer, directly interacting with the external environment and playing a critical role in various physiological and ecological functions. It is a primary target for antimicrobial agents and the immune system, requiring a delicate balance of strength and flexibility to provide protection while facilitating nutrient uptake, membrane vesicle exchange, and external signal reception (Gow and Lenardon, 2023). Previous studies have shown that polymeric particles ranging from 100 nm to 300 nm do not penetrate the cell walls of pathogenic fungi, such as *A. fumigatus* and *C. albicans* (Orasch et al., 2023). Thus, it can be proposed that nanoplastics exceeding 100 nm primarily interact with the surfaces of fungal cell walls, impacting the outer wall polymers and glycoproteins associated with the chitin and *β*-glucan-based inner wall skeleton. This interaction could disrupt spatial organization and dynamic regulatory functions, impairing the fungal ability to effectively respond to changes in growth conditions and potentially leading to toxicity. Interestingly, certain filamentous fungi may induce a “dusting effect,” wherein high concentrations of nanoplastics allow initially colonizing hyphae to adsorb or internalize these particles into vacuoles, subsequently metabolizing them into less toxic forms. This adaptive response may mitigate toxicity to later-growing hyphae, thereby promoting favorable conditions for fungal proliferation (Mafla-Endara et al., 2023). Previous research in bacterial systems has demonstrated that nanoplastics with diameters of 60 nm can penetrate cells, accumulating internally and enhancing the generation of ROS, which impose stress on bacterial cells and significantly inhibit their growth (Dai et al., 2022). It is plausible that nanoplastics of similar sizes may also compromise pathogenic yeast cells, such as *C. neoformans*, given that ROS can modulate the expression of virulence factors, including capsule and melanin production (Zaragoza et al., 2008; Momin and Webb, 2021). The toxicological impact of nanoplastics on fungal cells is multifaceted, encompassing redox imbalances, membrane damage, immune responses, and genotoxic effects, which can induce various forms of cellular injury simultaneously. Collectively, nanoplastics have the potential to significantly alter the physiological states of fungi, highlighting the urgent need for further research to elucidate the complex interactions and effects of nanoplastics on fungal ecology.

### 2.5 Potential effects of nanoplastics on fungal drug resistance

**Figure 2:**
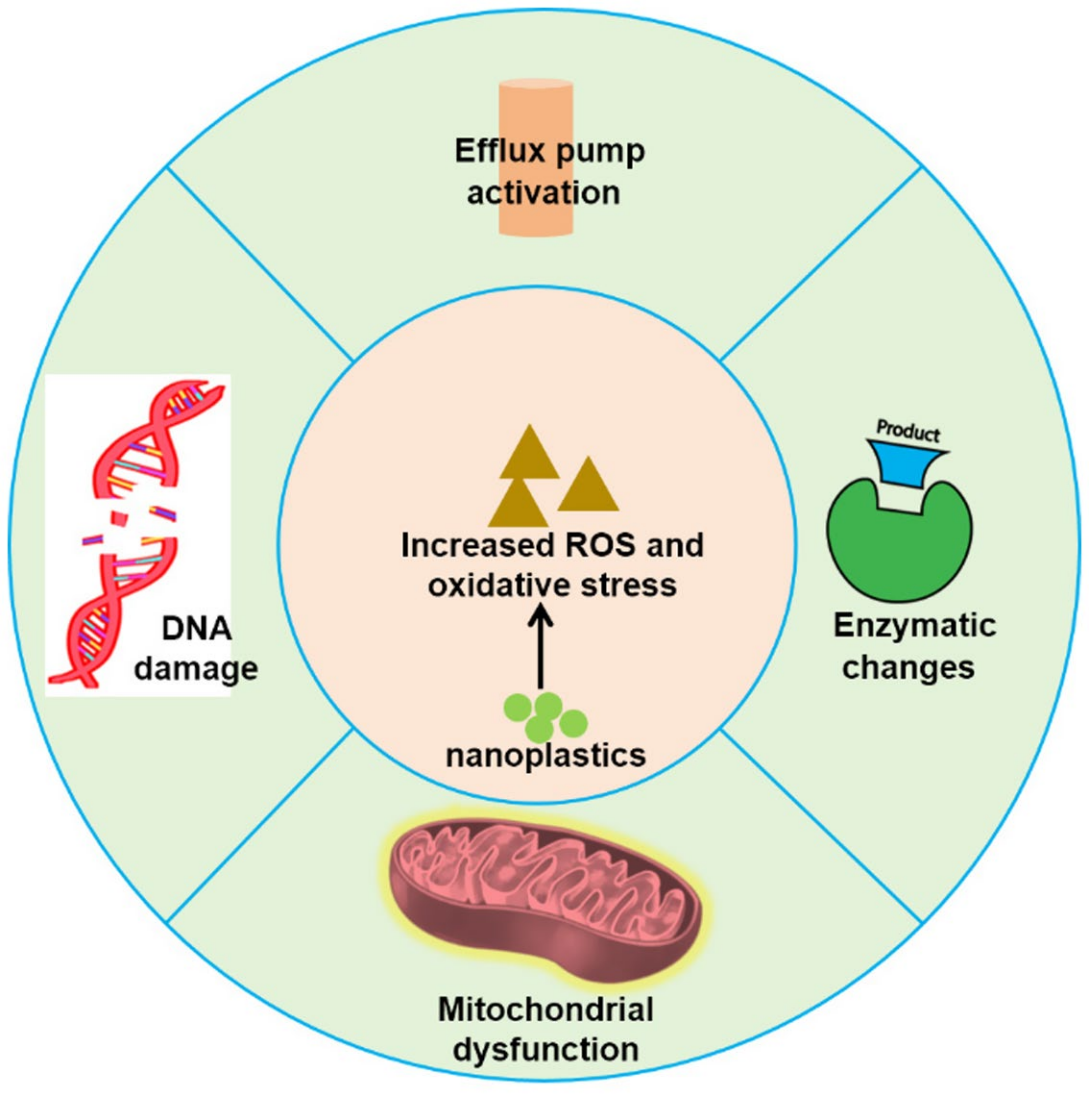
Schematic illustrating mechanisms of antifungal resistance potentially induced by nanoplastics.

The development of antifungal agents faces significant challenges due to the shared eukaryotic structures and metabolic pathways between humans and fungi, resulting in limited therapeutic options. Fungal infections are increasingly exhibiting resistance to conventional antifungal drugs, with the efficacy of existing treatments, such as azoles and polyenes, diminishing in clinical settings. According to data from the Centers for Disease Control and Prevention, drug-resistant fungal infections were responsible for at least 35,900 deaths in the United States in 2019 (Prevention, 2019). This resistance typically arises through natural selection, often driven by genetic mutations or gene transfer that confer additional resistant traits. Notably, fungi are sensitive to chemical toxicity and demonstrate rapid responses to environmental changes (Shruti et al., 2023). Recent studies suggest that nanoplastics may play a critical role in promoting antifungal resistance through several mechanisms. For instance, exposure to nanoplastics can trigger stress responses and adaptive mechanisms in fungi. In response to nanoplastic exposure, fungi may activate defense pathways, including oxidative stress responses and efflux pumps, which enhance their resistance to antifungal drugs. For example, *Lactarius deliciosus* exhibits oxidative stress in the presence of polystyrene, leading to increased secretion of organic acids and enhanced absorption of phosphorus and potassium, although growth is inhibited at high concentrations (Zhang and Gao, 2023). Moreover, exposure to nanoplastics has been shown to augment the secretion of extracellular enzymes in fungi, including *β*-glucosidase, glycine aminopeptidase, and phenol oxidase, thereby altering the community structure (Du et al., 2022). Similar observations in bacterial studies indicate that polystyrene exposure induces oxidative stress, leading to increased synthesis of glutathione and enhanced activity of the tricarboxylic acid (TCA) cycle, as well as of efflux pumps, which subsequently promote growth and resistance in *Escherichia coli* (Fang et al., 2023; Liu et al., 2023). Nanoplastics may also induce oxidative damage within fungal cells. For instance, low-density polystyrene has been shown to alter the membrane composition of *Trichoderma harzianum*, resulting in increased membrane permeability and enhanced activities of ROS, superoxide dismutase (SOD), and catalase (CAT) (Jasińska and Różalska, 2022). Recent findings from our research indicate that exposure to nanoplastics can induce ROS production in *C. neoformans*, disrupting normal cellular functions (unpublished data). Such oxidative damage may drive fungi to develop resistance through mutations or other adaptive changes that enhance their chances of survival. Moreover, fungal mitochondria play vital roles not only in cellular energy metabolism and oxidative stress responses but also in significantly influencing the activity and expression of drug efflux pumps (Black et al., 2021; Ma et al., 2025). Given that nanoplastics have been shown to induce mitochondrial damage in human cells (Lin et al., 2022), it is plausible to hypothesize that they could similarly affect mitochondrial function in fungi. Overall, the interaction of nanoplastics with fungi has the potential to significantly impact drug resistance (Figure 2). The multifaceted stress responses triggered by nanoplastics may not only enhance the ability of fungi to withstand antifungal agents but also promote the evolution of resistance mechanisms. These findings underscore the need for further research to elucidate the complexities of nanoplastic interactions and their implications for fungal pathogenicity and treatment strategies.

## 3 Conclusions and future perspectives

The intricate interactions between nanoplastics and fungi present a dual-edged sword for environmental science and public health. As emerging pollutants, nanoplastics have demonstrated significant effects on fungal physiology, including alterations in metabolic pathways, physiological responses, and even virulence factors. The ability of certain fungal species to degrade plastics offers promising avenues for bioremediation, yet the presence of nanoplastics complicates these interactions by influencing fungal physiological functions and potentially enhancing antifungal resistance mechanisms. This review highlights the necessity for a deeper understanding of the multifaceted relationships between nanoplastics and fungi. The evidence indicates that while fungi have the ability to degrade plastic, exposure to these pollutants may concurrently promote adaptations that enhance their resistance to antifungal agents. This paradox emphasizes the urgency of investigating the mechanisms underlying these interactions, as they could have far-reaching implications for both ecological health and clinical outcomes in fungal infections. Future research should focus on several key areas to elucidate the complex dynamics of nanoplastic-fungi interactions. Firstly, studies should aim to identify the specific molecular pathways activated in fungi upon exposure to nanoplastics, particularly regarding oxidative stress responses and enzymatic adaptations. Recent advancements in image analysis demonstrate how end-to-end image analysis and data fusion can provide high throughput and objective phenotyping (Iqbal et al., 2020; Iqbal et al., 2025), and can be adapted to quantify subtle, exposure-dependent morphological shifts in hyphae, spores, and biofilms. Secondly, the ecological impact of these interactions on fungal communities in various environments, including terrestrial and aquatic ecosystems, warrants further exploration. Long-term studies are essential to assess the implications of chronic nanoplastic exposure on fungal diversity and function. Additionally, advancing our understanding of the link between nanoplastic exposure and antifungal resistance mechanisms is critical. Investigating the potential for genetic mutations and horizontal gene transfer in fungi exposed to nanoplastics could provide insights into the development of resistance traits. This knowledge is particularly vital given the rising incidence of drug-resistant fungal infections that pose substantial public health threats. In conclusion, while the potential of fungi in bioremediation strategies remains promising, the challenges posed by nanoplastics necessitate a comprehensive investigation into their effects on fungal physiology and ecology. By addressing these research gaps, we can develop more effective strategies for managing plastic pollution and mitigating the associated risks to human health and the environment. Continued interdisciplinary collaboration will be crucial in paving the way for innovative solutions to combat the dual challenges of plastic pollution and fungal diseases.
